# Exosomes from IL-1β stimulated synovial fibroblasts induce osteoarthritic changes in articular chondrocytes

**DOI:** 10.1186/ar4679

**Published:** 2014-08-04

**Authors:** Tomohiro Kato, Shigeru Miyaki, Hiroyuki Ishitobi, Yoshihiro Nakamura, Tomoyuki Nakasa, Martin K Lotz, Mitsuo Ochi

**Affiliations:** Department of Orthopaedic Surgery, Graduate School of Biomedical Sciences, Hiroshima University, 1-2-3 Kasumi Minami-ku, Hiroshima, Japan; Department of Regenerative Medicine, Hiroshima University, Hiroshima, Japan; Department of Molecular and Experimental Medicine, The Scripps Research Institute, La Jolla, California, USA

## Abstract

**Introduction:**

Osteoarthritis (OA) is a whole joint disease, and characterized by progressive degradation of articular cartilage, synovial hyperplasia, bone remodeling and angiogenesis in various joint tissues. Exosomes are a type of microvesicles (MVs) that may play a role in tissue-tissue and cell-cell communication in homeostasis and diseases. We hypothesized that exosomes function in a novel regulatory network that contributes to OA pathogenesis and examined the function of exosomes in communication among joint tissue cells.

**Methods:**

Human synovial fibroblasts (SFB) and articular chondrocytes were obtained from normal knee joints. Exosomes isolated from conditioned medium of SFB were analyzed for size, numbers, markers and function. Normal articular chondrocytes were treated with exosomes from SFB, and Interleukin-1β (IL-1β) stimulated SFB. OA-related genes expression was quantified using real-time PCR. To analyze exosome effects on cartilage tissue, we performed glycosaminoglycan release assay. Angiogenic activity of these exosomes was tested in migration and tube formation assays. Cytokines and miRNAs in exosomes were analyzed by Bio-Plex multiplex assay and NanoString analysis.

**Results:**

Exosomes from IL-1β stimulated SFB significantly up-regulated *MMP-13* and *ADAMTS-5* expression in articular chondrocytes, and down-regulated *COL2A1* and *ACAN* compared with SFB derived exosomes. Migration and tube formation activity were significantly higher in human umbilical vein endothelial cells (HUVECs) treated with the exosomes from IL-1β stimulated SFB, which also induced significantly more proteoglycan release from cartilage explants. Inflammatory cytokines, *IL-6*, *MMP-3* and *VEGF* in exosomes were only detectable at low level. IL-1β, *TNFα MMP-9* and *MMP-13* were not detectable in exosomes. NanoString analysis showed that levels of 50 miRNAs were differentially expressed in exosomes from IL-1β stimulated SFB compared to non-stimulated SFB.

**Conclusions:**

Exosomes from IL-1β stimulated SFB induce OA-like changes both *in vitro* and in *ex vivo* models. Exosomes represent a novel mechanism by which pathogenic signals are communicated among different cell types in OA-affected joints.

## Introduction

Osteoarthritis (OA) is a highly prevalent disease in the middle-aged and elderly population worldwide. Pathogenesis has not been elucidated completely, and disease-modifying treatment and prevention are presently not available. OA risk factors include aging, acute or chronic mechanical stress, joint trauma, and metabolic disorders [1, 2]. These factors impair the homeostatic balance between cartilage extracellular matrix (ECM) degradation and repair. OA is a whole-joint disease and involves all joint tissues including cartilage, subchondral bone, menisci, ligaments and muscles. Joint inflammation at varying intensity is also present and contributes to the chronic joint tissue remodeling process and to pain, the main subjective symptom in OA patients [2–6]. The homeostatic balance of all joint tissues is regulated by intracellular molecules such as kinase cascades, autophagy, and transcription factors, epigenetic mechanisms, including miRNAs and by extracellular stimuli including cytokines, hormones and mechanical stress [7, 8].

Synovial inflammation and angiogenesis are important contributors to OA pathogenesis [3, 9–11]. Synovitis has been demonstrated to correlate with OA symptom severity, and hormonal factors such as cytokines, and chemokines are important for crosstalk among joint tissues [3, 12]. These mediators play a role in the development of inflammation and induce catabolic changes in joint tissues [3, 11, 13, 14]. Increased angiogenesis is also observed in OA-affected ligaments, menisci and subchondral bone [5, 6, 15].

Most nucleated cells release microvesicles (MVs), which range from 30 to 1,000 nm in diameter, and can be found in body fluids such as blood, urine, breast milk, and saliva [16–18]. MVs are also present in rheumatoid arthritis (RA) synovial fluids and can originate from granulocytes, monocytes, and other immune cells. These MVs modulate the release of chemokines and cytokines in synovial fibroblasts (SFB) [19–21]. The MVs derived from OA chondrocytes display annexins II, V, and VI, which play an important role in pathological mineral formation in OA [22–24]. Exosomes are one type of MV of endocytic origin released to the extracellular environment. These small particles, of about 30 to 200 nm, are derived from the fusion of multivesicular bodies to plasma membranes, and are morphologically distinct from larger secreted MVs [18, 25]. Exosomes can contain mRNA, microRNA and protein [26] and function in cell-to-cell communication as carriers of genetic information, and are associated with the pathogenesis of various diseases [19, 27]. Although release of MVs from SFBs has been reported [21, 28, 29], the effects of exosomes from OA synovial tissues on articular chondrocytes remain unknown. We hypothesized that exosomes function in a novel regulatory network that contributes to OA and elucidate in the present study the *in vitro* and *ex vivo* production, and the function of exosomes in the interaction between SFB and articular chondrocytes.

## Materials and methods

### Human tissues and cell culture

Studies were approved human subjects/ethics protocols by Scripps Research Institute Human Subjects Institutional Review Boards. Normal human knee synovial fibroblasts and chondrocytes were isolated from autopsy donors as leftover de-identified material and with no interactions with subjects, and therefore with no informed consent required. Human SFB and articular chondrocytes were cultured as described previously [30] in DMEM containing 10% FBS, and 1% penicillin/streptomycin at 37°C in a humidified atmosphere of 5% CO_2_. Experiments with SFB were carried out at passages 4 to 6. Experiments with normal human articular chondrocytes were carried out at passage 1.

### Preparation of conditioned medium and isolation of exosomes

Human SFB were plated at 2 × 10^5^ per well into 6-well plates with DMEM containing 10% FBS and 1% antibiotics at 37°C under 5% CO_2_. After reaching confluence, the cells were treated with DMEM containing recombinant human IL-1β (1 ng/mL; Pepro Tech NJ, USA), and incubated for 24 h. The SFB were washed three times with DMEM, and the medium was switched to fresh DMEM containing 10% FBS (2 mL). After incubation for 24 h, the SFB conditioned media (2 mL/well) were collected and cells were washed with PBS, and total RNA was isolated with TRIzol reagent (Life Technologies). The most widely accepted method for exosome isolation is ultracentrifugation. Recently, however, several alternate methods that are more standardized and efficient have been developed. In the present study, we used conventional ultracentrifugation and ExoQuick™ reagent kit (System Biosciences CA, USA) for isolation of exosomes [31–33], because these methods did not show a detectable difference in the type of exosomes obtained when applied to supernatants from cultured cells. The SFB-conditioned media (2 mL/well) were centrifuged for 15 minutes at 2380 × g to remove debris, and then further ultracentrifuged for 70 minutes at 110,000 × g (Optima TL ultracentrifuge, Beckman Coulter, Brea, CA, USA). The supernatants were collected as exosome-depleted conditioned media (CM-exo). The purified exosomes were resuspended in DMEM for direct use in subsequent experiments.

### Size distribution analysis by tunable resistive pulse sensor (TRPS)

Exosomes isolated by ultracentrifugation were resuspended in 100 mM KCl and 40 mM HEPES. The measurements of particle size and number were performed on an Izon qNano system by TRPS technology (Izon Science, Ltd Christchurch, New Zealand) as previously reported [34, 35].

### Immunoblotting for exosome markers

Exosomes and proteins isolated from cells were suspended in sample buffer solution (not containing 2-mercaptoethanol; Wako, Japan). Proteins were separated on Mini-PROTEAN TGX gels (Bio-Rad Laboratories, Hercules, CA, USA) and transferred to a polyvinylidene fluoride (PVDF) membrane by Trans-Blot Turbo™ transfer system (Bio-Rad Laboratories). Mouse anti-human CD9 (1:200, BD Biosciences), anti-CD81 (1:200, Santa Cruz Biotechnology, Inc), and anti-flotillin-1 (1:500, BD Biosciences) were used as the primary antibodies. Goat anti-mouse IgG-HRP (Santa Cruz, sc-2005) was used as the secondary antibody. Chemiluminescence signal was detected with Immno-enhancer (WAKO, Japan) using the ImageQuant LAS-4000 mini luminescent image analyzer (FUJIFILM, Japan).

### Cytokine levels in exosomes and conditioned medium from SFB

The measurements of cytokines (IL-1β, IL-6, TNFα, VEGF) and MMPs (MMP-3*,* -9*,* -13) within exosomes, CM-exo and conditioned media (CM) from SFB were performed by Bio-Plex suspension assay system (Bio-Rad Laboratories) as per the manufacturer’s protocol. Exosomes and CM-exo were isolated from conditioned medium by ultracentrifugation and the ExoQuick™ reagent kit.

### Treatment of normal human articular chondrocytes with SFB-derived exosomes

Human articular chondrocytes were seeded at 5 × 10^4^/ well into 24-well plates in DMEM containing 10% FBS, and cultured for 24 h. After the cells became confluent, exosome suspensions in DMEM and CM-exo were added. After incubation for 24 h, total RNA was extracted from chondrocytes using TRIzol reagent (Life Technologies).

### Quantitative real-time PCR

Complementary DNA (cDNA) was synthesized using 500 ng of total RNA with Superscript VILO kit (Invitrogen) according to the manufacturer’s protocol. Real-time PCR assay was performed using TaqMan Gene Expression Assays probes (Life Technologies) for *COL2A1* (Hs01064869_m1), *ACAN* (Hs00202971_m1), *MMP-13* (Hs00233992_m1), *ADAMTS-5* (Hs00199841_m1), *IL-6* (Hs00985639_m1), *MMP-3* (Hs00233962_m1), *IL-1β* (Hs00174097_m1), *TNFα* (Hs00174128_m1) and *GAPDH* (Hs02758991_m1). The expression levels for each gene were assessed relative to the expression of *GAPDH*. The ΔΔCt method was used for analysis of real-time PCR data.

### Proteoglycan release assay

Femoral heads were harvested from 4-week-old wild-type mice and incubated at 37°C for 72 h in 48-well plates. Each well contained 500 μl of DMEM with 10% FBS and 1% penicillin/streptomycin. The cartilage samples were washed three times, and cultured at 37°C for an additional 72 h in 500 μl of serum-free DMEM containing exosomes isolated by ExoQuick™ from SFB with IL-1β or without IL-1β. The assay was performed in at least three independent experiments with duplicate wells using SFB-derived exosomes. The concentration of the released glycosaminoglycan in the conditioned medium from cartilage was measured using the Blyscan Glycosaminoglycan assay kit (Biocolor UK) as per the manufacturer’s protocol.

### Endothelial cell migration assay

Human umbilical vein endothelial cells (HUVECs) were purchased from Lonza and cultured with endothelial basal medium-2 (EBM-2) (Lonza Basel, Switzerland). The migration of HUVECs was assessed using a Transwell Boyden Chamber (8-μm pore size) (BD Bioscience). HUVECs suspended in 0.5 mL of serum-free DMEM containing exosomes were added at 2 × 10^4^ cells/well to the 24-well upper chamber, and 0.75 mL of DMEM was added to the bottom well. After incubation for 4 h, the lower side of the filter was washed with PBS and fixed with 4% paraformaldehyde. The migrated cells were quantified as the number of cell nuclei stained with 4',6-diamidino-2-phenylindole (DAPI).

### Tube formation assay

HUVECs were pre-cultured overnight in DMEM with 0.25% FBS and then reseeded at a density of 2.5 × 10^4^ cells/well in 48-well plates pre-coated with Matrigel Matrix (BD Biosciences). HUVECs were treated with DMEM with 0.25% FBS containing, vascular endothelial growth factor (VEGF) (50 ng/mL as positive control), IL-1β (1 ng/mL) and exosomes for 4 h. Tube length was measured by means of Image J analysis of digital images.

### miRNA analysis in exosomes from SFB-conditioned medium

Exosomes were isolated from 15 mL of conditioned medium from SFB seeded at 1.5 × 10^6^ cells/15 cm plate by ultracentrifugation. Small RNA was purified from exosomes using the mirVana isolation kit (Life Technologies). The small RNA concentration and quality were determined by BioAnalyzer 2100 (Agilent Technologies, Santa Clara, CA, USA), and at least 5 ng RNA were then used as input for the nCounter Human miRNA Expression Assay kit (NanoString Technologies, Seattle, WA, USA). The miRNA expression profiles were analyzed according to manufacturer’s instructions.

### Statistical analysis

The data were analyzed using the Mann-Whitney *U*-test and Steel test to analyze statistical differences. Differences were considered statistically significant at a *P*-value <0.05 (*P <0.05; **P <0.01).

## Results

### Effect of IL-1β on gene expression in SFB

**Figure 1:**
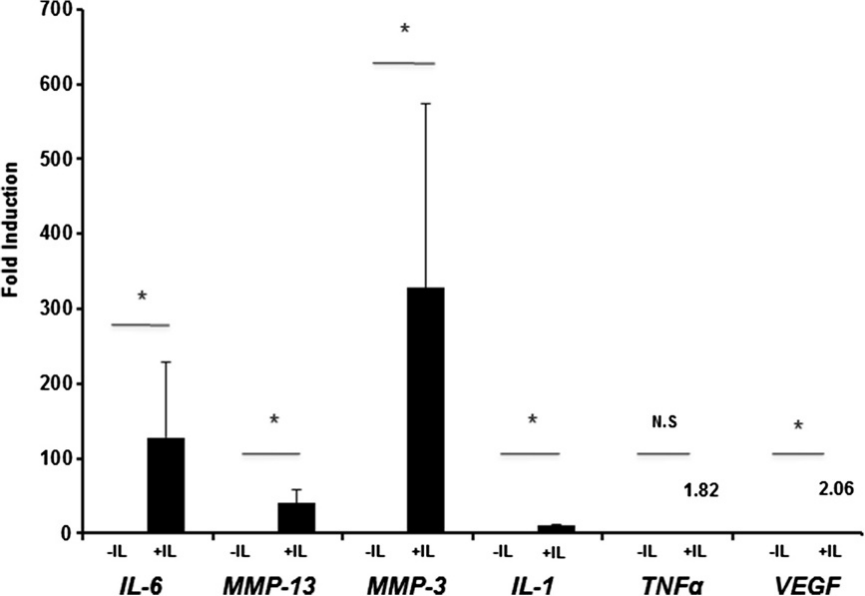
**IL-1β induced gene expression in synovial fibroblasts (SFB).** SFB (six different preparations from six different donors) were cultured with DMEM containing recombinant human IL-1β (1 ng/ml) for 24 h. The SFB were washed three times with DMEM, and the medium was switched to fresh DMEM containing 10% FBS. After incubation for 24 h, gene expression was analyzed by real-time PCR. Data are the means ± standard error of the mean. Comparison of mean values was performed by Mann-Whitney *U*-test; *P <0.05 versus non-stimulated SFB. NS, no significant change; *MMP*, matrix metalloproteinase; *VEGF*, vascular endothelial growth factor.

IL-1β is one of the critical mediators of OA, and IL-1β stimulation of SFB causes OA-like gene expression patterns [10, 11, 13]. To examine the gene expression changes by the IL-1β treatment in host cells (SFB) of exosomes, we performed real-time PCR for *MMP-3*, *MMP-13*, IL-1β, *IL-6*, *TNFα* and *VEGF* in SFB. In response to IL-1β stimulation, the expression of *MMP-3*, *MMP-13*, IL-1β, *IL-6*, and *VEGF* were significantly increased compared with non-stimulated SFB (Figure 1).

### Exosomes in IL-1β-stimulated SFB-conditioned medium

**Figure 2:**
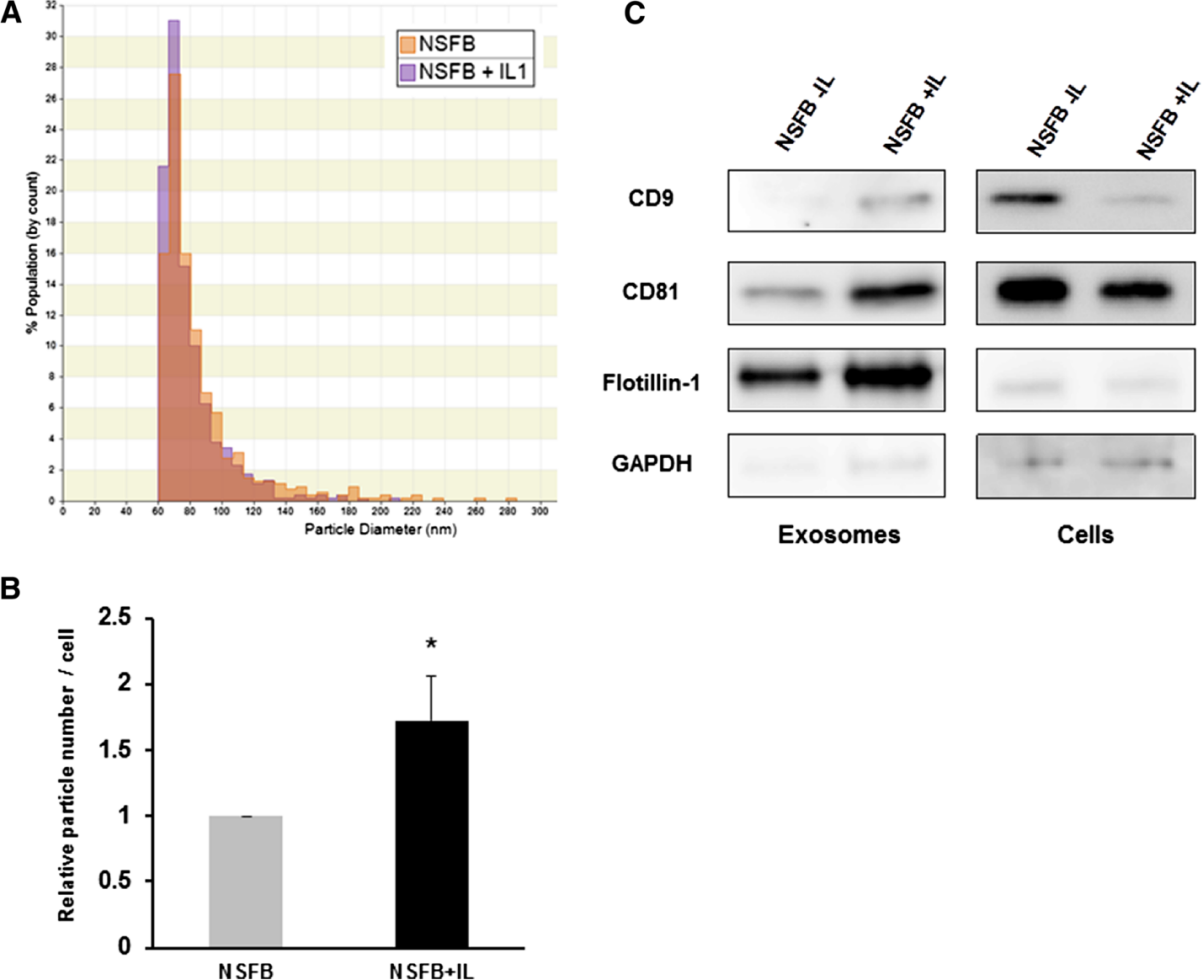
**Exosomes derived from synovial fibroblast (SFB)-conditioned medium. (A)** The size distribution of the particles in the ultracentrifugation pellet was measured with the qNano system. The major peak in particle size was at 60 to 80 nm and overall size distribution ranged between 60 and 200 nm. **(B)** The numbers of nanoparticles, including exosomes, isolated from IL-1β-stimulated SFB were significantly higher than the numbers in non-stimulated SFB (NSFB). Data are the means ± standard error of the mean. Comparison of mean values was performed by Mann-Whitney *U*-test; *P <0.05 versus non-stimulated SFB. **(C)** Immunoblotting of exosome markers, CD9, CD81, and flotillin-1, in isolated exosomes from IL-1β-stimulated SFB or non-stimulated SFB. Flot, flottilin-1; GAPDH, glyceraldehyde-3-phosphate dehydrogenase.

**Figure 3:**
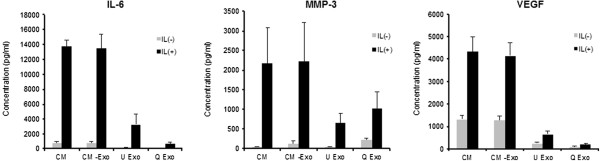
***IL-6*** **, matrix metalloproteinase (** ***MMP*** **)-** ***3*** **and vascular endothelial growth factor (** ***VEGF*** **) in exosomes derived from IL-1β-stimulated synovial fibroblasts (SFB).** The concentration of pro-inflammatory cytokines (IL-1β, *IL-6*, *TNFα*), angiogenic factor (*VEGF*) and cartilage degradation enzymes (*MMPs*) was measured in CM, exosomes, and CM-exo from IL-1β stimulated SFB using Bio-Plex assays. CM and CM-exo contained significant amounts of *IL-6*, *VEGF* and *MMP-3*. However, exosomes contained significantly lower *IL-6*, *VEGF* and *MMP-3* levels than CM or CM-exo. Data are the means ± standard error of the mean. CM, conditioned medium; CM-exo, exosome-depleted conditioned medium; U Exo, exosome isolated by ultracentrifugation method; Q Exo, exosome isolated by ExoQuick™ reagent.

To examine whether the secretion of exosomes from SFB is influenced by the IL-1β treatment, we analyzed the size and the number of isolated nanoparticles including exosomes from conditioned medium by ultracentrifugation. The size distribution of particles derived from SFB with IL-1β or without IL-1β was very similar, and the major peak in particle size was 60 to 80 nm and the overall size distribution ranged between 60 and 200 nm (Figure 2A). The numbers of nanoparticles including exosomes derived from IL-1β-stimulated SFB-conditioned medium were significantly increased compared with non-stimulated SFB-conditioned medium (Figure 2B). Several molecules, including the tetraspanins (CD9, CD63, CD81) and flotillin-1 are enriched in exosomes [25, 36]. We confirmed the presence of exosome marker proteins in conditioned medium from SFB (Figure 2C). Exosome markers (CD9, CD81, flotillin-1) in conditioned medium from IL-1β-treated SFB were also increased compared with SFB-conditioned medium without IL-1β (Figure 2C). Next, to examine cytokines and *MMPs* in exosomes from IL-1β-stimulated SFB, the concentration of cytokines was measured in CM, exosomes, and supernatant after ultracentrifugation (CM-exo) using Bio-Plex Multiplex assay. Inflammatory cytokines, *IL-6*, angiogenic factor, *VEGF* and *MMP-3* were included in CM and CM-exo from non-stimulated SFB, and were increased in CM and CM-exo from IL-1β-stimulated SFB. However, *IL-6*, *MMP-3* and *VEGF* levels in exosomes were lower than in CM and CM-exo (Figure 3). IL-1β, *TNFα*, *MMP-9* and *MMP-13* were not detectable in CM, CM-exo and exosomes.

### Exosomes from IL-1β-stimulated SFB induce OA-related gene expression patterns in articular chondrocytes

**Figure 4:**
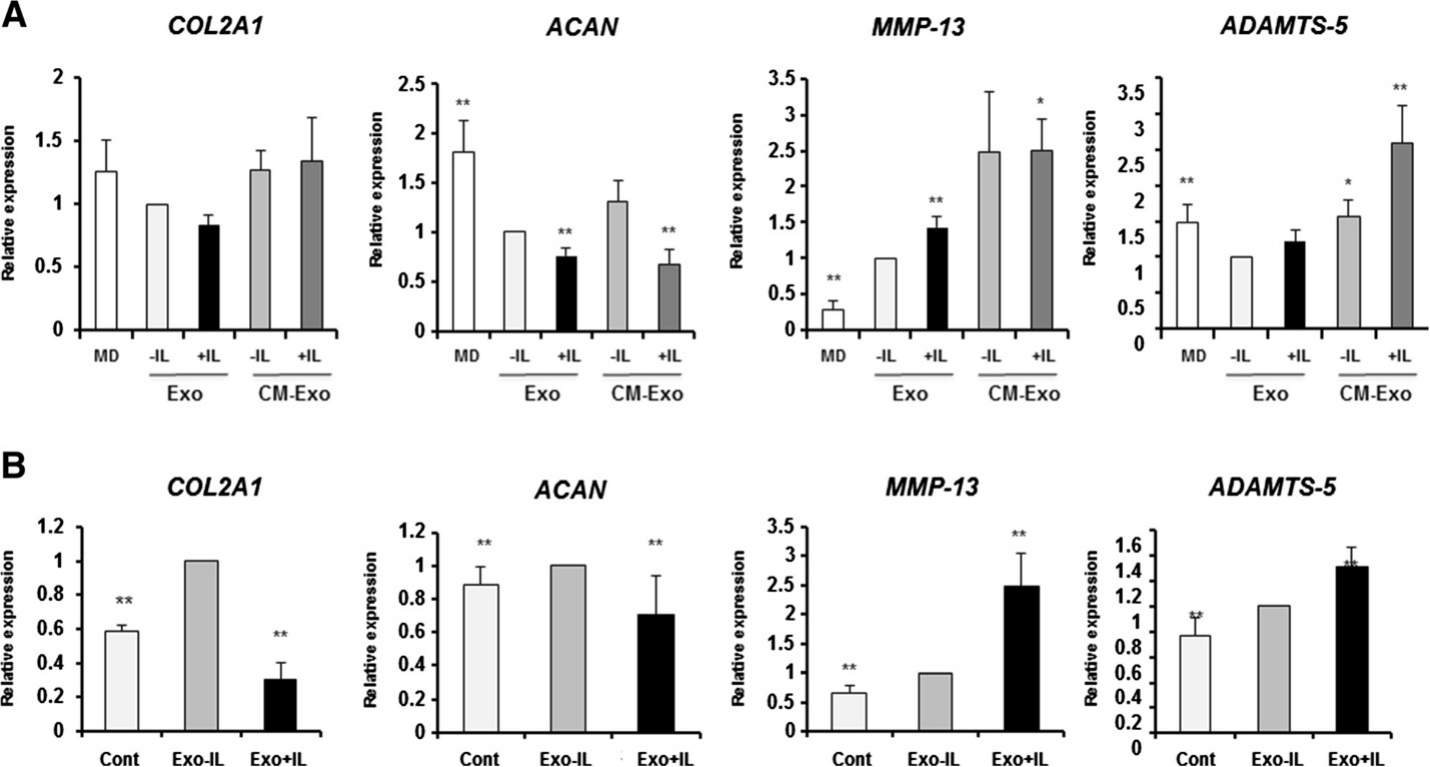
**The effect of IL-1β-stimulated synovial fibroblast (SFB)-derived exosomes on normal articular chondrocytes. (A)** Articular chondrocytes were treated with fresh-DMEM, exosomes from non-stimulated SFB or exosomes from IL-1β-stimulated SFB. Exosomes from SFB were isolated by ultracentrifugation from six different donors. The expression of osteoarthritis-related genes was analyzed by real-time PCR. Matrix metalloproteinase *(MMP*)-*13* was significantly upregulated, and *ACAN* significantly downregulated by exosomes from IL-1β stimulated SFB. Data are the means ± standard error of the mean (SEM). Comparison of mean values was performed by the Steel test; **P <0.01 versus Exo-IL. MD, fresh DMEM with 10% FBS; Exo -IL, exosomes from non-stimulated SFB; Exo + IL, exosomes from IL-1β stimulated SFB. **(B)** Exosomes were isolated from SFB-conditioned media (three separate cell preparations and experiments) using ExoQuick™ reagent. Exosomes from IL-1β-stimulated SFB significantly downregulated *COL2A1* and *ACAN* expression and upregulated *MMP-13* and *ADAMTS-5* expression in articular chondrocytes. An exosome layer was isolated using ExoQuick™ reagent from fresh-DMEM and was used as control. Data are the means ± SEM. Comparison of mean values was performed by the Steel test; **P <0.01 versus Exo -IL. Cont, control.

To elucidate the effects of exosomes from IL-1β-stimulated SFB, exosomes and CM-exo were added to articular chondrocytes. After incubation for 24 h with each medium, we examined the expression of OA-related genes by real-time PCR. Exosomes and CM-exo from IL-1β-stimulated SFB significantly upregulated *MMP-13* expression compared with exosomes from non-stimulated SFB. In contrast, the expression of *ACAN* was significantly downregulated (Figure 4A). *TNFα* expression was significantly increased by exosomes from IL-1β-stimulated SFB, but not by CM-exo from IL-1β-stimulated SFB (data not shown). The changes of *MMP-13*, and *ADAMTS-5* expression in articular chondrocytes with CM-exo from IL-1β-stimulated SFB were significantly greater than those observed with exosomes (Figure 4A). The expression of *VEGF* was not increased in articular chondrocytes treated with exosomes and CM-exo from IL-1β-stimulated SFB (data not shown). These findings suggest that the exosomes from IL-1β stimulated SFB induce expression of OA-related genes in articular chondrocytes. Isolated exosomes fraction by ExoQuick™ also significantly induced OA-related gene expression patterns in articular chondrocytes (Figure 4B).

### Proteoglycan release from cartilage explants by exosomes from IL-1β-stimulated SFB

**Figure 5:**
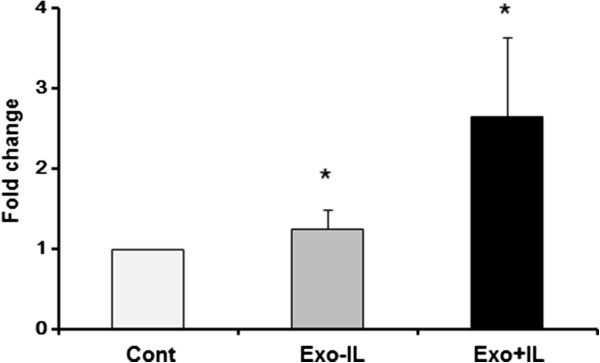
**Effect of synovial fibroblast (SFB)-derived exosomes in cartilage explants.** Mouse femoral head cartilage explants cultured with exosomes from non-stimulated SFB or IL-1β-stimulated SFB. Exosomes were isolated from SFB-conditioned medium using ExoQuick™ reagent. Proteoglycan release into the conditioned medium from cartilage was analyzed. Exosomes from IL-1β-stimulated SFB induced significantly more proteoglycan release than exosomes from non-stimulated SFB. Data are the means ± standard error of the mean. Comparison of mean values was performed by the Steel test; *P <0.05 versus control (Cont). Cont, isolated exosomes from fresh-DMEM with 10% FBS; Exo -IL-1β, exosomes from non-stimulated SFB; Exo + IL-1β, exosomes from IL-1β-stimulated SFB.

To determine whether IL-1β-stimulated SFB-derived exosomes induce cartilage catabolic changes, we quantified proteoglycan loss from mouse femoral head cartilage explants. Exosomes from IL-1β stimulated SFB significantly increased proteoglycan release compared with exosomes from non-stimulated SFB (Figure 5).

### SFB derived exosomes induce angiogenesis

**Figure 6:**
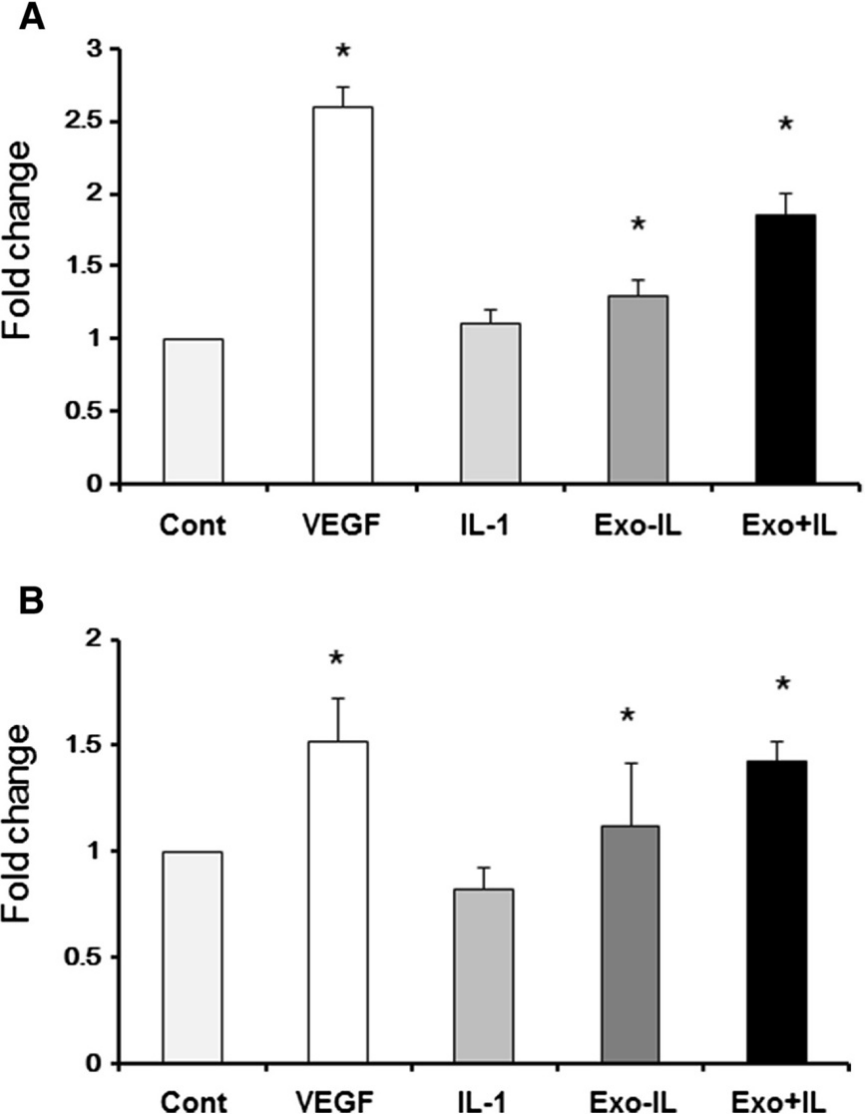
**Effects of synovial fibroblast (SFB)-derived exosomes on angiogenic activity.** SFB-derived exosomes were tested on human umbilical vein endothelial cells (HUVEC) for stimulation of migration and tube formation. Exosomes were isolated by ExoQuick™ reagent. **(A)** Migration activity was significantly higher in HUVEC treated with the exosomes from IL-1β-stimulated SFB as compared to PBS and the exosomes from SFB without IL-1β. HUVEC migration was not induced in HUVEC by IL-1β alone. Data are the means ± standard error of the mean (SEM). Comparison of mean values was performed by the Steel test; *P <0.05 versus control. **(B)** Tube length was significantly greater in HUVEC incubated with the exosomes from IL-1β-stimulated SFB than in HUVEC incubated with PBS and the exosomes from non-stimulated SFB. Data are the means ± SEM. Comparison of mean values was performed by the Steel test; *P <0.05 versus control. Cont, isolated exosomes from fresh-DMEM with 10% FBS; Exo -IL-1β, exosomes from non-stimulated SFB; Exo + IL-1β, exosomes from IL-1β stimulated SFB; Cont, control.

To examine the effects of exosomes from IL-1β-stimulated SFB on angiogenic activity, migration and tube formation assays were performed with HUVECs. Migration activity in HUVECs treated with exosomes from IL-1β-stimulated SFB was significantly higher than with exosomes from non-stimulated SFB (Figure 6A). The same pattern of differences was observed in the tube formation assay (Figure 6B). Addition of IL-1β did not increase migration and tube formation (Figure 6A, B). These results suggest that exosomes from IL-1β-stimulated SFB contain angiogenic signals.

### miRNA in exosomes from OA-like SFB

**Figure 7:**
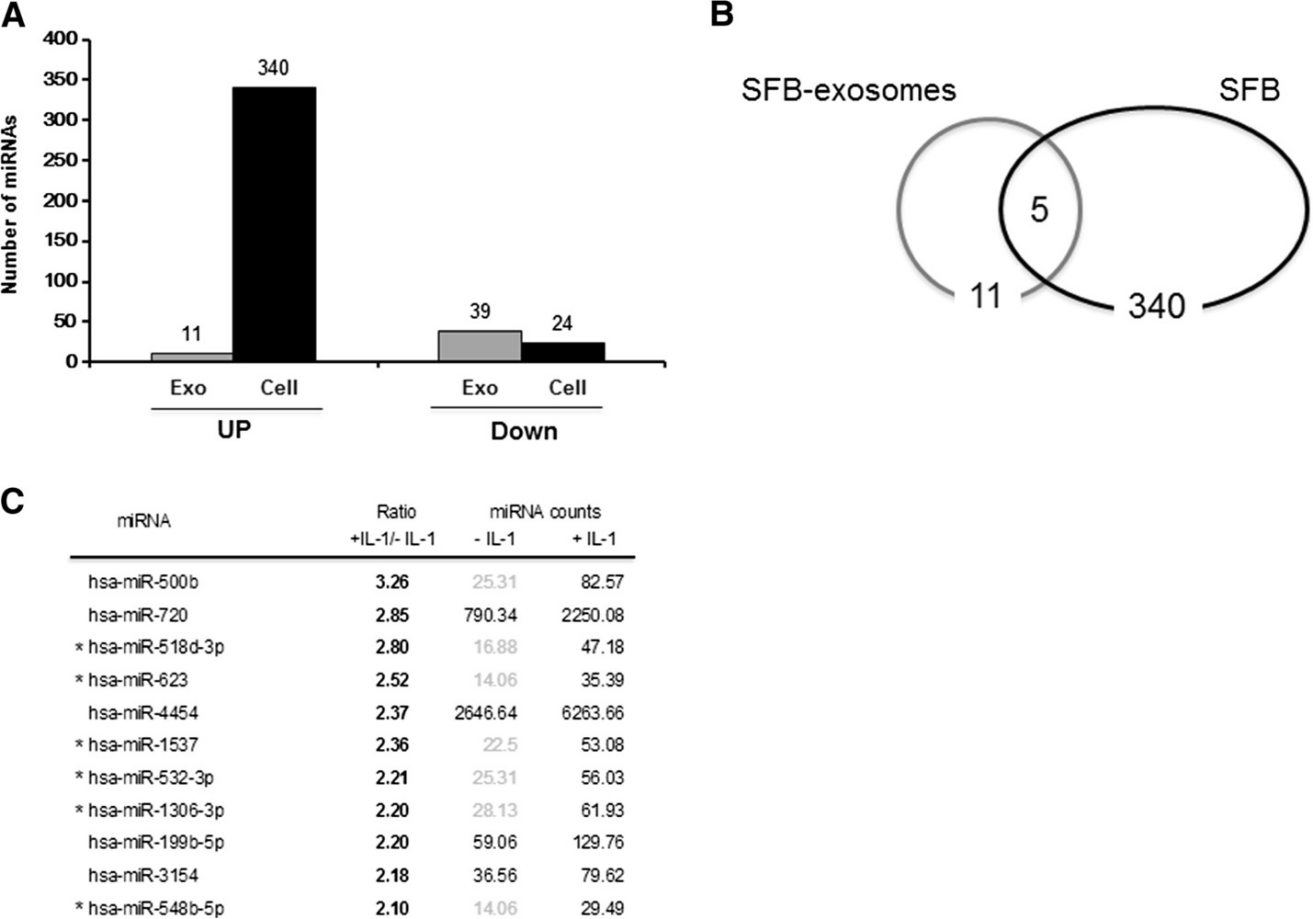
**Identification of miRNAs in exosomes from synovial fibroblasts (SFB). (A)** Expression profiling of 800 human miRNA in SFB with IL-1β or without IL-1β, and exosomes from IL-1β-stimulated or non-stimulated SFB: 340 miRNA were upregulated and 24 miRNA downregulated in IL-1β-stimulated SFB, and 11 miRNA were upregulated and 39 miRNA downregulated in exosomes from IL-1β-stimulated SFB. **(B)** Venn diagram comparing miRNA in IL-1β-stimulated SFB and exosomes from IL-1β-stimulated SFB. Five miRNA commonly presented in IL-1β-stimulated SFB and exosome from IL-1β-stimulated SFB, and six miRNA selectively presented in exosomes. **(C)** Differentially upregulated miRNA (2-fold difference) are shown as the ratio of exosomes from IL-1β-stimulated SFB to exosomes from non-stimulated SFB. Values for miRNA in exosomes from IL-1β stimulated SFB and exosomes from non-stimulated SFB are miRNA levels (nanostring code count). *miRNAs were not expressed in SFB.

To further examine whether miRNAs in exsomes induce OA-like changes, we characterized miRNA in exosomes using the NanoString system, comparing IL-1β-stimulated SFB with non-stimulated SFB. The IL-1β treatment had a major effect on miRNA expression in SFB, increasing the expression of 340 miRNA and decreasing 24 of them: 11 miRNA in exosomes were increased and 39 were decreased by IL-1β (Figure 7A). Among them, upregulated miRNA in both IL-1β-stimulated SFB and their exosomes were five miRNA, and six miRNA selectively presented in exosomes (Figure 7B). Differentially expressed miRNA in exosomes are shown as the ratio of miRNA in exosomes from IL-1β-stimulated SFB to miRNA in exosomes from non-stimulated SFB (Figure 7C).

## Discussion

This study focused on exosomes as a new secretion and communication factor in OA. IL-1β is one of the most prominent mediators of cartilage degradation and joint inflammation [11, 13, 37]. Therefore, we used IL-1β to simulate an OA environment in the SFB and their conditioned medium. Recently, extracellular vesicles, including exosomes, have attracted attention as new players in cell-to-cell communication [26, 38–40]. Exosomes may directly stimulate target cells through receptor-mediated interactions or may transfer from the host cell to the recipient cell various bioactive molecules such as proteins, mRNA and miRNA [27]. Exosomes are defined as small vesicles between 30 and 100 nm in diameter [18]. Although they are distinguished from other MVs based on their size, some differences are still unclear. Exosomes are formed intracellularly within multivesicular bodies and they have common surface components, such CD9, CD63, CD81, and flotillin-1 [25, 36]. Although MVs (100 to 1,000 nm in diameter) are larger than exosomes, they are shed from the plasma membrane surface. MVs that are 200 to 700 nm in diameter and are released from lymphocytes and monocytes induce *MMPs* and cytokine expression by SFB [21]. The exosomes isolated from SFB-conditioned media ranged in size mainly between 60 nm and 120 nm, and expressed exosome markers, including CD9, CD81 and flotillin-1. Furthermore, the release of exosomes was increased by IL-1β stimulation to SFB. Although the mechanisms of exosome release from cells remain incompletely understood, the release of exosomes has been shown to be affected by the pathological environment and culture condition [41–43]. These reports suggest that the OA pathological tissue environment regulates the release of exosomes from joint cells, including SFB. In this regard, exosomes may also reflect the specific disease environment. Therefore, exosomes have potential as diagnostic markers, and also offer insight into new regulatory pathways of pathogenesis.

*MMPs*, inflammatory cytokines and growth factors cause OA-like changes in joint tissues [10, 11, 13]. IL-1β-stimulated SFB and SFB from osteoarthritic joints can secrete a variety of cytokines and growth factors *in vivo* and *in vitro*, including *MMPs*, *IL-6* and *VEGF*, which is a major factor in inflammation and angiogenesis [11, 13, 37]. Indeed, we showed that exosomes from IL-1β stimulated SFB increased the expression of *MMP-3*, *IL-6,* and *VEGF. MMP-3*, *IL-6* and *VEGF* were present in CM and CM-exo. However, their levels were significantly lower in exosomes than in CM and CM-exo. After ultracentrifugation for isolation of exosomes, the supernatants were collected as CM-exo. Indeed, exosome markers such as CD9 and flotillin-1 were not detected in CM-exo (data not shown). These results indicate that almost *IL-6* and *VEGF* were contained in CM-exo after exosomes isolation by ultracentrifugation method. Although CM-exo induced the OA-like gene expression patterns in articular chondrocytes, exosomes from IL-1β-stimulated SFB also induced the OA-like gene expression patterns, and they stimulated proteoglycan release from cartilage explants. Therefore, the OA-like changes may be not only attributable to cytokines and growth factors, but also to exosomes. The exosomes from IL-1β-stimulated SFB induced expression of OA-related genes, especially in articular chondrocytes. These results suggest that other factors, including miRNA in exosomes, mediate OA-like changes. Furthermore, we showed that IL-1β-stimulated SFB-derived exosomes induced angiogenic activities such as migration and tube formation in HUVEC. Increased angiogenesis occurs in most tissues in osteoarthritic joints, including synovium, menisci, ligaments and subchondral bone and inhibition of angiogenesis has been shown to be effective in models of OA [3, 9–11]. Therefore, exosomes may promote increased angiogenesis in OA joint tissues. Taken together, SBF-derived exosomes may function as a novel regulatory factor that contributes to OA pathogenesis via OA-like signals such as catabolic and angiogenic signals to joint tissues.

miRNA are small noncoding RNA of 1 to 25 nucleotides that repress the translation of and/or cleave mRNA by partially base-pairing with the untranslated regions and/or coding regions of their target transcripts [44]. Therefore, miRNA can silence the expression of multiple genes [44]. Several miRNA, such as miR-140, are associated with OA pathogenesis [7, 45, 46]. Although previous studies have focused on intracellular miRNA, miRNA have recently been identified in several extracellular compartments. Exosomes contain proteins, mRNA and miRNA, and protect miRNA from RNase [26, 36], and miRNA can be transferred between tissues and even between individuals via biological fluids [16, 26]. However, it is reported that the extracellular miRNA in blood plasma and cell culture is independent from exosomes and is bound to Ago2 protein, a part of RNA-induced silencing complex [47, 48]. Thus, we may have identified extracellular miRNA rather than miRNA in exosomes. IL-1β stimulation of SFB increased the amount of released exosomes from SFB. Furthermore, the IL-1β stimulation altered the miRNA expression pattern not only in SFB but also in released exosomes. Although most of the upregulated miRNAs in IL-1β-stimulated SFB were also upregulated in their exosomes, several miRNA were identified only in exosomes. This result suggests a selective mechanism of miRNA release from cells. Indeed, although several studies reported these findings [26, 38–40, 49, 50], this mechanism is not still well-understood. Among upregulated miRNA (>2-fold) in exosomes from IL-1β-stimulated SFB, six miRNA were selectively present in exosomes. MiRNA in condition-specific exosomes may function as a unique set of miRNA. These results suggest that miRNA may not only function as regulatory molecules within cells such as SFB and chondrocytes, but also as components of exosomes contributing to OA pathogenesis by mediating cell-cell and tissue-tissue communication in osteoarthritic joints. Future studies need to determine the amount of exosomes released from non-OA- and OA-derived tissues and from cells, and the amount present in synovial fluid, and to identify the messenger molecules in exosomes that mediate the observed effects on chondrocytes.

## Conclusions

The present results show that SFB secrete exosomes, and IL-1β-stimulated SFB release increased the numbers of exosomes compared with non-stimulated SFB. miRNA are selectively presented in the exosomes. Exosomes from IL-1β-stimulated SFB lead to OA-like changes in gene expression patterns in human articular chondrocytes, and cartilage degradation. In addition, these exosomes stimulated angiogenic activity. SFB-derived exosomes may be involved in OA pathogenesis by stimulating gene expression in articular chondrocytes, and promoting angiogenesis. Our observations suggest that not only established signaling molecules, such as cytokines and hormones, but also exosomes including miRNA, as mediators of communication among different joint cells and tissues play an important role in OA pathogenesis as a new regulatory mechanism.

## Abbreviations

*ACAN*: aggrecan
*ADAMTS*: a disintegrin and metalloproteinase with thrombospondin motifs
CM: conditioned media
CM-exo: exosome-depleted conditioned media
Cont: control
DMEM: Dulbecco’s modified Eagle’s medium
EBM-2: endothelial basal medium-2
ECM: extracellular matrix
EXO: exosome
FBS: fetal bovine serum
Flot: flottilin-1
HUVEC: human umbilical vein endothelial cells
*IL*: interleukin
*MMP*: matrix metalloproteinase
MV: microvesicle
NSFB: normal human synovial fibroblast
OA: osteoarthritis
OASFB: osteoarthritis synovial fibroblast
PBS: phosphate-buffered saline
SFB: synovial fibroblast
*TNF*: tumor necrosis factor
*VEGF*: vascular endothelial growth factor.
